# The Bottlenecks in Translating Placenta-Derived Amniotic Epithelial and Mesenchymal Stromal Cells Into the Clinic: Current Discrepancies in Marker Reports

**DOI:** 10.3389/fbioe.2020.00180

**Published:** 2020-03-13

**Authors:** Seyyed-Hadi Ghamari, Mohsen Abbasi-Kangevari, Tahereh Tayebi, Soheyl Bahrami, Hassan Niknejad

**Affiliations:** ^1^Department of Pharmacology, School of Medicine, Shahid Beheshti University of Medical Sciences, Tehran, Iran; ^2^Student Research Committee, Social Determinants of Health Research Center, Shahid Beheshti University of Medical Sciences, Tehran, Iran; ^3^Ludwig Boltzmann Institute for Experimental and Clinical Traumatology in AUVA Research Center, Vienna, Austria

**Keywords:** amniotic epithelial cells, cell heterogeneity, cross-contamination, epithelial to mesenchymal transition, isolation protocol, mesenchymal stromal cells, passage number, simultaneous isolation

## Abstract

Placenta-derived amniotic cells have prominent features for application in regenerative medicine. However, there are still discrepancies in the characterization of human amniotic epithelial and mesenchymal stromal cells. It seems crucial that the characterization of human amniotic membrane cells be investigated to determine whether there are currently discrepancies in their characterization reports. In addition, possible causes for the witnessed discrepancies need to be addressed toward paving the way for further clinical application and safer practices. The objective of this review is to investigate the marker characterization as well as the potential causes of the discrepancies in the previous reports on placenta-derived amniotic epithelial and mesenchymal stromal cells. The current discrepancies could be potentially due to reasons including passage number and epithelial to mesenchymal transition (EMT), cell heterogeneity, isolation protocols and cross-contamination, the region of cell isolation on placental disk, measuring methods, and gestational age.

## Introduction

Human amniotic membrane has increasingly attracted the attention of basic and clinical scientists in recent years as a promising source of cells for regenerative medicine. It is a thin avascular membrane which forms a fluid-filled sac enclosing the fetus, which consists of an epithelial layer, a basal lamina, and an avascular mesenchymal layer which includes a compact layer, a fibroblast layer and a spongy layer (Gupta et al., 2015). The epithelial layer consists of flat, cuboidal and columnar epithelial cells which are in contact with amniotic fluid. Attached to the epithelial layer is the basal lamina composed of collagen, fibronectin, and laminin (Hilmy et al., 2018). The mesenchymal layer is connected to the basal lamina and includes fibroblast-like mesenchymal stromal cells, and a defined population of HLA-DR-expressing cells with macrophage-monocyte phenotypic (Magatti et al., 2008). A spongy layer consisting of loosely arranged collagen fibers separates the mesenchymal layer and the chorion. The number of human amniotic epithelial cells (hAECs) is four to eight times greater than human amniotic mesenchymal stromal cells (hAMSCs), depending on the gestational age (Ochsenbein-Kolble et al., 2003).

Extensive research has focused on placenta-derived amniotic membrane as a potential cost-effective unlimited source of cells which could be obtained less invasively compared to procedures like bone marrow biopsy. With parental consent, the placenta could be obtained from elective caesarian sections which have lower risk of microbial contamination compared with vaginal births (Adds et al., 2001). In addition, obtaining amniotic membrane does not require any embryos to be destroyed and is associated with limited ethical considerations compared with the use of embryonic stem cells (Parolini et al., 2008). Human amniotic cells could be used rather safely due to lack of tumorigenicity (Liu et al., 2018; Abbasi-Kangevari et al., 2019). Furthermore, they do not express MHC class II surface markers and have low immunogenicity (Peric et al., 2018). Therefore, they could be used as an allogenic transplant which highlights their potential application among the elderly who do not possess sufficient pools of stem cells for autograft transplantation (Ahmed et al., 2017). Although characteristics of the cells could be affected according to the culture condition such as the components of culture medium, hAECs can sufficiently be expanded under certain culture condition and maintain their reproducible biologic characteristics including expressing major pluripotent genes as well as embryonic stem cell specific surface markers in the subculturing process (Evron et al., 2011). Like many stem cells, human amniotic cells could also be cryopreserved which makes them suitable for banking due to low costs in terms of expense, time, and human resources (Murphy et al., 2014; Yazdanpanah et al., 2015). Moreover, they have ease of isolation and self-renewal capacities which make them a promising option for applications in regenerative medicine. Human amniotic cells have the potential to differentiate into all the three germ layers including endoderm, mesoderm, and ectoderm; i.e., hepatocytes, pancreatic cells (Wei et al., 2003), cardiomyogenic (Miki, 2011), chondrogenic, osteogenic, adipogenic (Shu et al., 2011; Topoluk et al., 2017; Ghasemzadeh et al., 2018), and neurogenic cell lines (Portmann-Lanz et al., 2006).

There is a rapidly growing body of literature on clinical trials which investigate the potential application of hAECs and hAMSCs in the clinic, considering their immunomodulatory features (Yamahara et al., 2019), wound healing promotion (Prakoeswa et al., 2018), prevention and treatment of pulmonary disorders (Moodley et al., 2010; Baker et al., 2019), treatment of premature infants with bronchopulmonary dysplasia (Lim et al., 2018). In addition, they are being investigated in recruiting or non-recruiting clinical trials in Asherman's Syndrome (The Second Affiliated Hospital of Chongqing Medical University and Shanghai iCELL Biotechnology Co., Ltd, Shanghai, China, 2017) and Graft-vs. -Host Disease (PUPS and Shanghai iCELL Biotechnology Co., Ltd, Shanghai, China, 2018).

An international workshop was held in 2008 which focused on the structure of amnion and discussed isolation, characterization, and differentiation protocols for hAECs, and hAMSC, as well as the immunomodulatory properties, *in vitro* and *in vivo* preclinical studies, and cell banking strategies for these cell populations (Parolini et al., 2008). However, there are still discrepancies in the recent reports on the characterization of human amniotic epithelial and mesenchymal stromal cells. Possible causes for the witnessed discrepancies among the characterization reports need to be addressed toward paving the way for further clinical application and safer practices. The objective of this review is to investigate the marker characterization as well as the potential causes of the discrepancies in the previous reports on placenta-derived amniotic epithelial cells and mesenchymal stromal cells.

## Discrepancies in Characterization of Human Amniotic Cells

**Figure 1:**
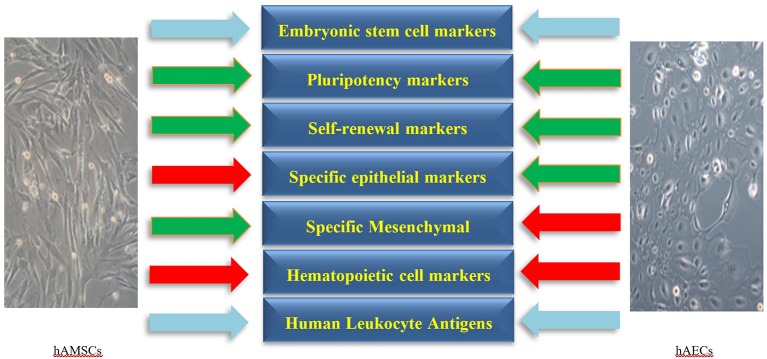
The main negative (red arrows) and positive (green arrows) markers on human amniotic epithelial (hAECs) and mesenchymal stromal cells (hAMSCs). The expression of Human Leukocyte Antigens and embryonic stem cell markers on hAECs and hAMSCs is variable (sky-blue arrows). Specific markers for each category are presented in more details in Table 1.

Human amniotic cells including hAECs and hAMSCs are derived from the epiblast and hypoblast layers of amnion after 8 days of fertilization, respectively. These cells form a heterogeneous population of pluripotent, multipotent, progenitor, and mature cells (Miki and Strom, 2006; Rennie et al., 2012) which are characterized by the presence of embryonic stem cell and pluripotency markers. Moreover, the expression of epithelial, mesenchymal, and Human Leukocyte Antigens (HLAs) varies among hAECs and hAMSCs. As the amniotic membrane is adjacent to the chorion, the isolated cells of amnion origin need to be negative for hematopoietic markers to rule out hematopoietic cell contamination. In addition, hAECs and hAMSCs express various lineage-associated markers, which represents their potential to differentiate to several cell lineages as progenitor cells. Characterization markers of hAECs and hAMSCs are presented in the following categories (Figure 1, Table 1).

**Table 1:** Characterization of human amniotic membrane-derived cells.

	**Human amniotic epithelial cells (hAECs)**	**Human amniotic mesenchymal cells (hAMCs)**	**References**
**Phenotype of cells**	**Flat, cuboidal, and columnar epithelial cells**	**Plastic-adherent, spindle-shaped cells**	
**Positive markers during passages**			
Embryonic stem cell, self-renewal, and pluripotency markers	TRA1-60^†^^×^, TRA1-81^†^^×^, SSEA-3^*^, SSEA-4^*^ ^×^, OCT4^*^ ^×^, Nanog^*^ ^×^, SOX-2^*^ ^×^, SOX17^*^ ^×^, KLF-4^*^ ^×^, c-MYC^*^ ^×^, REX-1, CFC-1, DPPA-3, PROM-1, PAX-6, FOXD3, GDF3, TfE3	TRA1-60^†^^×^, TRA1-81^†^^×^, OCT3, OCT4^*^ ^×^, Nanog^*^ ^×^, SOX-2^*^ ^×^, SOX17^*^ ^×^, KLF-4^*^ ^×^, c-MYC^*^ ^×^	Miki et al., 2005; Ilancheran et al., 2007; Bilic et al., 2008; Ge et al., 2012; Roubelakis et al., 2012; Zhou et al., 2013; Koike et al., 2014; Chen et al., 2015; Garcia-Castro et al., 2015; Samsonraj et al., 2017
Epithelial cell markers	CK-1, CK-2, CK-3, CK-4, CK-5, CK-6, CK-7, CK-8, CK-10, CK-13, CK-14, CK-15, CK-16, CK-19, CA-125, MUC-16, CD326^×^^*^(EpCAM), CD324^*^ (E-Cadherin), CD73	CD324^*^, CD326^×^^*^(EpCAM)	Nanbu et al., 1989; Diaz-Prado et al., 2011; Pratama et al., 2011; Caruso et al., 2012; Paracchini et al., 2012; Centurione et al., 2018
Mesenchymal cell markers	CD73^*^, CD271^*^, CD24^†^^*^, CD90^†^^*^, CD133^*^^†^, CD44^†^^×^	CD73^*^, CD271^*^, CD24^*^, CD90^*^, CD133^*^, CD105^×^	Soncini et al., 2007; Murphy et al., 2010; Roubelakis et al., 2012; Iaffaldano et al., 2013; Sivasubramaniyan et al., 2013; Koike et al., 2014; Spitzhorn et al., 2017; Schmelzer et al., 2019
Human leukocyte antigens	HLA-A^*^, HLA-B^*^ HLA-C^*^, HLA-DR^*^	HLA-A^*^, HLA-B^*^, HLA-C^*^	Bilic et al., 2008; Fatimah et al., 2010; Koike et al., 2014; Magatti et al., 2015, 2016; Pogozhykh et al., 2015
Specific cell lineage and functional markers	Apo-D, A2B5^*^, MMP-1, PDGF Receptor ß (CD140b), Musashi-1, Nestin^*^, Vimentin^*^ ^×^, PSA-NCAM, β-tubulin-III, Catecholamine, Norepinephrine, Dopamine, DOPAC, Choline acetyltransferase (ChAT), Acetylcholine, GATA-4^*^, Hepatocyte nuclear factor-3ß, AFP^×^, Albumin^*^, Glucose-sensing molecule (GLUT-2), Insulin, RCI^*^, Neurofilament proteins^*^, MAP2 kinase^*^, Microtubule-associated protein 2^*^ (MAP2), Glial fibrillary acidic protein^*^, CNPase^*^, Myelin basic protein^*^, Galactocerebroside^*^, Atrial myosin light chain- 2^*^ (MLC-2A), Ventricular myosin light chain- 2^*^ (MLC-2V), Nkx 2.5^*^, α-actinin, collagen type II^*^, Osteocalcin^*^, Osteopontin^*^, ALP^*^, Type I collagen	CD133, Nestin^*^, Albumin^*^, α-fetoprotein^×^ (α-FP), Cytokeratin 18 (CK18), α1-Antitrypsin (α1-AT), Hepatocyte nuclear factor 4α (HNF4α), PDX-1, RCI^*^, A2B5^*^, Neurofilament proteins^*^, MAP2 kinase^*^, Microtubule-associated protein 2^*^ (MAP2), Glial fibrillary acidic protein^*^, CNPase^*^, Myelin basic protein^*^, Galactocerebroside^*^, Atrial myosin light chain- 2^*^ (MLC-2A), Ventricular myosin light chain- 2^*^ (MLC-2V), GATA-4^*^, Nkx 2.5^*^, α-actinin, collagen type II^*^, Osteocalcin^*^, Osteopontin^*^, ALP^*^, type I collagen^*^	Sakuragawa et al., 1996, 2000, 2004; Elwan and Sakuragawa, 1997; Kakishita et al., 2000; Takahashi et al., 2001, 2002; Wei et al., 2003; Portmann-Lanz et al., 2006; Kim et al., 2007; Gomez Dominguez, 2008; Kong et al., 2008; Parolini et al., 2008; Tamagawa et al., 2008; Manuelpillai et al., 2011; Miki, 2011; Niknejad et al., 2012; Alcaraz et al., 2013; Fatimah et al., 2013; Koike et al., 2014; Garcia-Lopez et al., 2015; Sarvandi et al., 2015; Bollini et al., 2018; Centurione et al., 2018; Liu et al., 2018; Maymo et al., 2018
Other markers	CD1b,CD9^*^, CD10, CD13^†^, CD24, CD26, CD29^*^,CD31, CD34, CD46, CD49a, CD49b, CD49c, CD49d, CD49e^*^, CD49f, CD55, CD58, CD59, CD63, CD77, CD81,CD83, CD91, CD95, CD98, CD104, CD109, CD117^×^^*^, CD 133, CD142, CD144,CD146, CD147, CD151, CD164,CD166^*^, CD227, ABCG2/BCRP	CD9^*^, CD13^*^, CD27^†^, CD29^*^, CD31, CD49e^*^, CD54, CD166^*^, CD117^*^ ^×^, CD349, Vimentin^*^ ^×^, STRO-1, BMP-4	Gomez Dominguez, 2008; Fatimah et al., 2010; Murphy et al., 2010; Pratama et al., 2011; Zhou et al., 2013; Koike et al., 2014; Pozzobon et al., 2014; Magatti et al., 2015
**Negative markers during passages**	CD11,CD14^*^, CD31^*^, CD62, CD349, HLA-A2, VWF	CD3, CD14^*^, CD34, CD45, CD324 (E-cadherin), HLA-DR^×^, HLA-DP, HLA-DQ	Miki et al., 2005; Portmann-Lanz et al., 2006; Ilancheran et al., 2007; Wolbank et al., 2007; Bilic et al., 2008; Murphy et al., 2010; Pratama et al., 2011; Magatti et al., 2012, 2016; Koike et al., 2014; Alikarami et al., 2015; Si et al., 2015; Phermthai et al., 2017; Samsonraj et al., 2017

* *Expressed on both hAECs and hAMSCs*.

† *Low expression*.

× *Discrepancy witnessed*.

### Embryonic Stem Cell, Self-Renewal, and Pluripotency Markers

Although the specific phenotypic features of hAECs and hAMSCs including plastic-adherence, microscopic shape of the cells and the potential to form colony-forming units is of value in cell characterization, their identification essentially relies on characterization using markers of embryonic stem cell, self-renewal, and pluripotency which remains challenging. The expression of specific surface markers of undifferentiated embryonic stem cells including Tumor Rejection Antigen (TRA) 1-60, 1-81, Stage Specific Embryonic Antigens (SSEA)-3, SSEA-4, Octamer-Binding Transcription Factor 4 (OCT-4), Nanog, SOX-2, Kruppel-like factor 4 (KLF-4), REX-1, CFC-1, Developmental Pluripotency Associated 3 (DPPA-3), Prominin 1 (PROM-1), Paired Box Protein 6 (PAX-6), Forkhead box D3 (FOXD3), Growth differentiation factor-3 (GDF3), TFE3, and c-MYC has been studied among hAECs and hAMSCs. It has been shown that SSEA-3 and SSEA-4 are present on 9% and 44% of hAECs, respectively (Miki et al., 2005). Another study also detected SSEA-3 and SSEA-4 on hAECs, but in a different quantity: 40% and 97%, respectively (Zhou et al., 2013). While almost 10% of hAECs express TRA1-60 and TRA1-81 on their surface (Miki et al., 2005), a study suggested that TRA1-60 could be a ubiquitous marker for isolating stem cells from heterogeneous amnion epithelial cells (Koike et al., 2014). A study suggested that hAECs, but not hAMSCs, express TRA1-60, TRA1-81, SSEA-3, and SSEA-4 (Zhou et al., 2013). Consistently, it has been reported that low or no protein levels of TRA1-60 and TRA1-80 were detected on hAMSCs (Roubelakis et al., 2012; Koike et al., 2014). A study reported that only a small percentage of hAMSCs expressed SSEA-4 at P0, which decreased during P2 (Magatti et al., 2016). In contrast, it has been reported that SSEA-4 was expressed among 43% of hAMSCs (Koike et al., 2014). There are also other studies that reported the expression of SSEA-3 and SSEA-4 among hAMSCs (Roubelakis et al., 2012; Samsonraj et al., 2017).

The expression of pluripotency markers including Oct3/4, Nanog and KLF-4 is higher among hAMSCs than other sources of mesenchymal stem cells (Koike et al., 2014). In a study, reverse transcriptase-PCR analysis exhibited transcripts of Oct-3/4 among both hAMSCs and hAECs; however, immunocytochemistry confirmed translation into Oct-3/4 protein by a sub-population of hAECs, but not among hAMSCs (Bilic et al., 2008). There are studies which reported pluripotency of hAMSCs with a high expression of pluripotency-specific genes including Nanog and OCT-4 among other pluripotency genes (Miki et al., 2005; Ge et al., 2012). It has been reported that the expression of pluripotency markers including SOX-2, Nanog, KLF-4, and c-MYC decreased during the culture of mesenchymal stromal cell (Chen et al., 2015).

It has been reported that hAECs express molecular markers that are known to be essential for self-renewal and pluripotency including OCT-4, Nanog, SOX-2, KLF-4 and REX-1 at first culture (P_0_) and also during passages (Miki et al., 2005; Garcia-Castro et al., 2015). Term freshly isolated (P_0_) hAECs express mRNA of OCT-4, SOX-2, CFC-1, Nanog, DPPA-3, PROM-1, and PAX-6, while the mRNA of the pluripotency markers FOXD3 and growth differentiation factor 3 (GDF3) were not detected among hAECs population (Ilancheran et al., 2007). A study quantified the expression of pluripotency surface and molecular markers in the first culture of hAECs and through the passages by quantitative real-time PCR and immunostaining. They reported that 10%, 17%, and 52% of hAECs were positive for OCT4, SOX2, Nanog at P_0_, respectively. Therefore, it could be suggested that at least 10% of the isolated epithelial cells population from human amniotic membrane have pluripotency features which makes it an appropriate source for cells. The percentage of hAECs that expressed OCT-4, SOX-2, and Nanog did not change significantly during passages one to four (P_1−4_). Furthermore, they measured the presence of E-cadherin (CD324) which was expressed by human pluripotent stem cells and demonstrated that almost 100% of hAECs were positive for E-cadherin. Protein expression of KLF-4 and transcription factor binding to IGHM enhancer 3 (TfE3) was high in P_0_ culture of hAECs and increased significantly during the second passage (P_2_) (Garcia-Castro et al., 2015). Therefore, although both hAECs and hAMSCs express embryonic stem cell, self-renewal, and pluripotency markers, the level of marker expression remains variable.

### Epithelial Cell Markers

Studies indicate that hAECs express epithelial specific markers including pan-cytokeratin (CK) 1, 2, 3, 4, 5, 6, 7, 8, 10, 13, 14, 15, 16, 19, Carbohydrate Antigen (CA) 125, Mucin (MUC) 16, EpCAM (CD326), and E-Cadherin (Nanbu et al., 1989; Diaz-Prado et al., 2011; Pratama et al., 2011; Caruso et al., 2012). It has been reported that more than 98% of hAECs express E-Cadherin and CD73 (Centurione et al., 2018). Amniotic epithelial cells showed high expression of integrin and increasing expression of CK3 and CK19 during serial passages; however, the expression of CK1 and CK14 decreased during serial passages which suggests that hAECs may be differentiated during passages (Fatimah et al., 2010). Several studies reported negative expression of epithelial cell markers by hAMSCs (Koike et al., 2014; Si et al., 2015; Phermthai et al., 2017; Magatti et al., 2018); however, a study reported low level of expression of E-Cadherin (Iaffaldano et al., 2013). Another study on hAMSCs reported that CK19 was strongly positive at passage 0 and 1 and decreased to zero level at passage 6 (Gomez Dominguez, 2008). E-Cadherin has been used as a marker to prove epithelial contamination in the characterization of isolated hAMSCs (Mariotti et al., 2008).

### Mesenchymal Cell Markers

There are studies which reported that hAMSCs expressed mesenchymal markers including vimentin, CD73, CD90, and CD105. Vimentin remained strongly positive at passage 0, 1 and 6 among hAMSCs (Roubelakis et al., 2012; Koike et al., 2014; Spitzhorn et al., 2017). The expression of CD73, CD90, and CD105 increased during passages and more than 95% of hAMSCs expressed CD73, CD90, and CD105 from P2 to P4 (Samsonraj et al., 2017). A recent study confirmed that more than 90% of hAMSCs were positive for CD90 and CD73; however, they reported that CD105 was expressed only by 4% of hAMSCs (Schmelzer et al., 2019). Mesenchymal cell-related markers including CD24, CD133 and CD271 were positive on hAMSCs; however, the expression of CD271 remained controversial. A study identified mesenchymal stromal cells via CD271, while another study reported that only bone marrow-derived mesenchymal stromal cells among all types of mesenchymal stromal cells expressed CD271 (Soncini et al., 2007; Sivasubramaniyan et al., 2013). In addition, a study reported a lack of expression of CD271 on hAMSCs (Iaffaldano et al., 2013), while another study indicated that 50% of hAMSCs expressed CD271 (Koike et al., 2014).

Moreover, hAECs express mesenchymal stromal cell and mesenchymal cell-related antigens. Almost 69% and 38% of hAECs express CD73 and CD271, respectively (Zhou et al., 2013; Koike et al., 2014). The expression of CD24, CD90, and CD133 by hAECs was also observed (Fatimah et al., 2010; Zhou et al., 2013; Koike et al., 2014); however, <1% of hAECs expressed CD44 (Zhou et al., 2013; Koike et al., 2014). The expression of mesenchymal markers including vimentin and CD140-B increased on hAECs during passages, which is possibly suggestive of the epithelial-mesenchymal transition potential of hAECs (Miki and Strom, 2006; Pratama et al., 2011).

### Hematopoietic Cell Markers

Almost none of the hAECs and hAMSCs express hematopoietic markers including CD14, CD34, and CD45 (Murphy et al., 2010; Koike et al., 2014; Si et al., 2015; Phermthai et al., 2017; Samsonraj et al., 2017). However, a study detected CD14, CD34 and CD45 on 10%, 3% and 17% of hAMSCs, respectively. There might be small colonies of CD14 and CD45-positive mesenchymal cells at P0, which could be attributed to human amniotic mesenchymal tissue cell contamination (Magatti et al., 2012, 2015, 2016).

### Human Leukocyte Antigens

Despite hAECs and hAMSCs possess different morphology and marker expression, they have the same potential of modulating immunoreactions (Wolbank et al., 2007). The immunologic profiles of hAECs and hAMSCs showed that they both expressed very low levels of HLA A, B and C immediately after isolation (P0); however, the level of these antigens on hAECs increased significantly by P2 (Fatimah et al., 2010; Pogozhykh et al., 2015). Furthermore, a study reported that freshly isolated hAECs may express some type-I-MHC antigens including HLA-A, HLA-B or HLA-C as evaluated by a pan antibody against HLA-ABC (Magatti et al., 2015). It has been observed that hAECs displayed negligible expression of type II MHC including HLA-DR, DP and DQ (Fatimah et al., 2010), while the expression of HLA-DR on hAMSCs remained controversial. Although some studies reported negative expression of HLA-DR by hAMSCs, a study indicated that 14% of hAMSCs were HLA-DR-positive (Bilic et al., 2008; Koike et al., 2014; Magatti et al., 2016). Magatti et al. reported small groups of HLA-DR-positive hAMSCs at P0, as well. However, the level of HLA-DR deceased during P2 compared to P0 (Magatti et al., 2016).

It is worth mentioning that the low-level expression of type I HLA and the lack of expression of type II HLA markers alongside the expression of immune privileging HLA-G and co-stimulatory molecules including CD40, CD40 ligand, CD80 (B7-1), and CD86 (B7-2) on hAECs and hAMSCs demonstrates their potential immunomodulatory value in transplantation, which may enable them to be applied across the major histocompatibility barrier (Lefebvre et al., 2000; Chang et al., 2006; Banas et al., 2008; Parolini et al., 2008; Lim et al., 2013; Peric et al., 2018). In addition, hAECs and hAMSCs neither express the Programmed Death-1 (PD-1), an inhibitory receptor that is generally expressed on activated T and B cells, nor Programmed Death Ligand 1 and 2 (PDL-1/2) (Okazaki and Honjo, 2007; Wu et al., 2014).

### Specific Cell Lineage Markers

Amniotic membrane cells include a heterogeneous population of stromal cells and precursor cells. These cells are clonogenic and their primary cultures could differentiate into specific cells of three germ lineages which express various markers of ectodermal, endodermal, and mesodermal cells (Miki et al., 2005).

Since hAECs and hAMSCs express specific markers of ectodermal cells derived neuronal, oligodendrocytes, and glial cells, they have shown potential for treating central nervous system disorders as well as having the capacity to produce and secrete neurotransmitters. In 2008, Sakuragawa et al. reported for the first time that these cells expressed high levels of neural cells specific antigens including RCI, A2B5, vimentin, Neurofilament proteins, microtubule-associated protein (MAP) 2, and MAP2 kinase. It has been reported that hAECs had a high expression level of neural specific markers including Musashi-1, Nestin, vimentin, PSA-NCAM, and β-tubulin-III (Kong et al., 2008). There are studies which reported the production of neurotransmitters related proteins in hAECs, including catecholamine, norepinephrine, dopamine, 3,4-Dihydroxyphenylacetic acid (DOPAC), choline acetyltransferase (ChAT) and acetylcholine (Elwan and Sakuragawa, 1997; Sakuragawa et al., 1997; Kakishita et al., 2000). They also showed high expression of glial cells specific markers including glial fibrillary acidic protein, CNPase, myelin basic protein, and Galactocerebroside (Sakuragawa et al., 2004; Portmann-Lanz et al., 2006; Kim et al., 2007; Tamagawa et al., 2008). In addition, it has been reported that fibroblast markers including matrix metallopeptidase 1 (MMP-1) and ApoD were expressed in freshly isolated hAECs; however, the expression level was low in comparison to adult fibroblasts (Koike et al., 2014). PDGF Receptor ß (CD140b) expression on freshly isolated hAECs (P0) alongside vimentin expression is negative; however, 90% of hAECs are positive for CD140b at passage 6 (P6) (Parolini et al., 2008; Miki, 2011; Alcaraz et al., 2013; Centurione et al., 2018). Hepatic markers including GATA-4 and hepatocyte nuclear factor-3ß were detected in amnion-derived epithelial cells by RT-PCR (Wei et al., 2003; Bollini et al., 2018). The expression of the hepatic markers and proteins including alpha fetoprotein (AFP) and albumin were high on both mRNA and protein levels among freshly isolated hAECs (Takahashi et al., 2001, 2002; Liu et al., 2018; Maymo et al., 2018). Amniotic epithelial cells strongly expressed glucose-sensing molecule GLUT-2 on mRNA levels which is characteristic for beta cells of pancreas and hepatocytes (Wei et al., 2003; Garcia-Lopez et al., 2015). Furthermore, albumin synthesis and excretion by hAECs have been detected by immunostaining and enzyme-linked immunoassay (Sakuragawa et al., 2000). It has been shown that undifferentiated hAMSCs expressed genes associated with hepatocytes and pancreatic cells including albumin, AFP, CK18, α1-Antitrypsin (α1-AT), hepatocyte nuclear factor 4α (HNF4α) and the pancreatic lineage-associate marker PDX-1 (Manuelpillai et al., 2011; Sarvandi et al., 2015).

Although hAMSCs originate from avascular stromal layer of the amniotic membrane, they express endothelial and angiogenic markers including von Willebrand Factor (vWF), platelet/endothelial cell adhesion molecule (PECAM-1/CD31), vascular endothelial growth factor (VEGF), vascular endothelial growth factor receptor-2 (VEGFR-2), Fibroblast Growth Factor (FGF) and angiopoietin-1 (Fatimah et al., 2013). It has been reported PECAM-1, bFGF, eNOS, VEGF, VEGFR-2, and vWF expressions decreased during passage of hAMECs; however, angiopoietin-1 expression was increased. On the other hand, endothelial markers including PECAM-1 (CD31), E-selectin (CD62e), and vWF were almost negative on hAECs (Gomez Dominguez, 2008).

It has been reported that hAECs and hAMSCs express cardiac-specific genes including atrial myosin light chain- 2 (MLC-2A), ventricular myosin light chain- 2 (MLC-2V), GATA-4, and Nkx 2.5 in media supplemented with ascorbic acid. In addition, it has been observed that they expressed α-actinin, a mature cardiomyocyte marker, which was detected by Immunohistochemical analysis (Miki et al., 2005). A summary of characterization markers of human amniotic membrane-derived cells is presented in Table 1.

## Causes of Discrepancies in Reports on Phenotype and Markers

**Figure 2:**
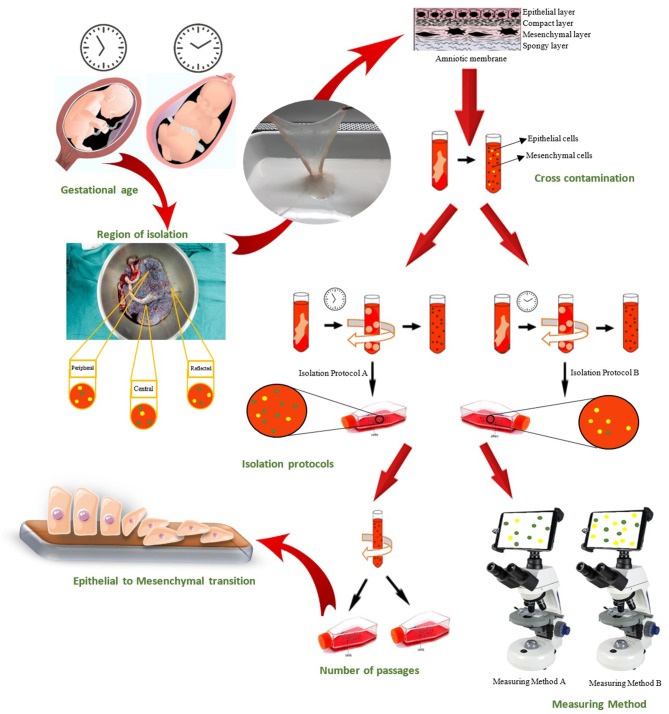
Causes of witnessed discrepancies in characterization of human amniotic epithelial and mesenchymal stromal cells could be categorized in seven groups: Gestational age of placenta, Region of cell isolation on placenta, Cross-contamination of amniotic epithelial and mesenchymal stromal cell, Isolation protocol, Epithelial-to-mesenchymal transition of hAECs, Passage number of isolated cells, and Measuring methods that used for characterization.

Extensive research has focused on the possible applications of amnion-derived cells in the clinic. However, the discrepancies in the reports on characterization of hAECs and hAMSCs need to be considered to further pave the way for their clinical utilization. There are various possible causes for the current discrepancies in reports on characterization markers of hAECs and hAMSCs, which are mentioned here (Figure 2).

### Passage Number and Epithelial to Mesenchymal Transition

Several studies indicate that the number of passages influence the marker expression on hAMSCs and hAECs. Like mesenchymal stromal cells from other sources, hAMSCs do not express HLA-DR in earlier passages (Pittenger et al., 1999; Covas et al., 2003; In 't Anker et al., 2003; D'Ippolito et al., 2004; Gotherstrom et al., 2004; Lee et al., 2004; Portmann-Lanz et al., 2006; Koike et al., 2014). However, a study on amniotic membranes without culturing reported expression of HLA-DR on hAMSCs (Kubo et al., 2001). Another study reported the expression of MHC antigens in the early passages of hAMSCs which disappeared in later passages (Kim et al., 2007). A suggested explanation for the reported discrepancy among studies has been the multiple passages of the cells which diminished the expression of HLA-DR (Kim et al., 2007).

Along with alterations in surface marker expressions, hAECs' morphology gradually changes toward mesenchymal phenotype over several passages (Bilic et al., 2008) and transmission electron microscopic studies of hAMSCs suggested an epithelial–mesenchymal hybrid phenotype (Pasquinelli et al., 2007). These observations are interpreted as epithelial to mesenchymal transition (EMT) (Portmann-Lanz et al., 2006; Bilic et al., 2008; Pratama et al., 2011). EMT occurs in the natural process of placental development, where extravillous cytotrophoblasts transit to mesenchymal phenotype which allows them to migrate and infiltrate into the maternal decidua and vessels. It has been shown that hAECs are initially negative for the mesenchymal marker vimentin; however, they become vimentin positive during the EMT process (Alcaraz et al., 2013). Along with the rise in vimentin, fibronectin, and *N*-cadherin levels (Guarino et al., 2009; Tsuji et al., 2009), E-cadherin protein levels which are high at early passages become undetectable in later passages which confirms EMT among the amniotic cells (Alcaraz et al., 2013). Although some studies consider CD73 to be a mesenchymal-specific marker (Brown et al., 2019), others introduce it as an epithelial-specific marker (Centurione et al., 2018). A recent study has suggested that CD73 mechanistically promotes the expression of EMT-associated genes, which could shed more light on the witnessed discrepancy regarding the role of CD73 in characterization of hAMSCs and hAECs (Lupia et al., 2018).

### Cell Heterogeneity

Cell heterogeneity could be another explanation for the current discrepancies in reports on characterization of hAECs and hAMSCs. Amniotic membrane cell populations are seemingly heterogeneous and thus may differ in their phenotypic and molecular properties (Roubelakis et al., 2012; Niknejad et al., 2016). A study reported that among mesenchymal cells isolated from chorion, placental decidua, and amniotic membrane, the highest heterogeneity could be from those isolated from amniotic membrane. Miki et al. suggested that there might be various lineage-committed multipotent cells in the population of hAECs (Miki, 2011). The heterogeneity of hAMSCs and hAECs has been confirmed by immuno-phenotypic and morphological analysis (Araujo et al., 2017). Various levels of expression of pluripotency and proliferation markers in hAECs including OCT-4, CD117, SOX-2, a-fetoprotein, CREB, and p- CREB; various proliferation capability; and osteogenic potential could indicate their heterogeneity. In addition, Sakuragawa et al. demonstrated that hAECs express phenotype of both neural and glial cells (Sakuragawa et al., 1996). Heterogeneity also applies to functional molecules as well as growth factors secreted by hAECs and hAMSCs. Several studies have shown that amniotic membrane cells are capable of expressing erythropoietin (Ogawa et al., 2003), pulmonary surfactant (Lemke et al., 2017), dopamine (Niknejad et al., 2012), catecholamine (Sakuragawa et al., 1996) activin (Koyano et al., 2002), brain-derived neurotrophic factor, neurotrophin-3, and nerve growth factor (Uchida et al., 2000; Jin et al., 2015), all of which are involved in fetal early development.

### Isolation Protocols and Cross-Contamination

Isolation of cells of human amniotic membrane could become challenging due to its histologic feature. hAECs and hAMSCs are located within layers adjacent to each other, which increases the risk of simultaneous isolation and cross-contamination. Cross-contamination is defined as the contamination of hAECs with hAMSCs and vice versa rather than the pure population of the desired cells. Although the expression of CD117 in hAECs is almost always positive, it has been reported that hAMSCs are negative or weakly positive for CD117 (Bilic et al., 2008; Roubelakis et al., 2012; Magatti et al., 2016). While CD44 is a characteristic marker of mesenchymal stromal cells, a study reported that CD44 is a positive marker for the isolated hAECs (Roubelakis et al., 2012; Insausti et al., 2014). Furthermore, several studies for isolation of hAECs yielded cells that were positive for CD105, which is a defined mesenchymal stromal cells marker (Miki et al., 2010; Murphy et al., 2010, 2014; Tabatabaei et al., 2014). In a protocol for the isolation of hAECs more than 56 percent of cells were CD105 positive (Gramignoli et al., 2016). Simultaneous isolation of cells could be a possible cause for the witnessed discrepancy.

Several protocols have been proposed for isolation of hAECs and hAMSCs with a wide range of cells yielded, viability, and purity (Motedayyen et al., 2017; Araujo et al., 2018; Kitala et al., 2018). However, the protocol of isolation affects phenotype and function of the yielded cells. A study compared two protocols and reported that the isolated hAMSCs had some differences (Diaz-Prado et al., 2011). Soncini's protocol yielded hAMSCs by cutting amniotic membrane into small pieces; enzymatic digestion for 7 min by Dispase; resting period for 10 min; second enzymatic digestion by collagenase and DNase for 3 h, centrifuge at 200 g for 10 min; and culture (Soncini et al., 2007). Alviano's protocol is consisted of mincing; two enzymatic digestion of amniotic membrane by Trypsin/EDTA, collagenase and DNase for 15 and 5 min, respectively; centrifuge at 200 g for 10 min; and culture in DMEM with 20% FBS and 1% penicillin–streptomycin (P/E) (Alviano et al., 2007). The expression of CD117 marker was significantly higher in hAMSCs isolated by Soncini's protocol, which suggested that this protocol isolated more progenitor cells than Alviano's protocol. In addition, the expression of SSEA-4 and STRO-1 were higher among hAMSCs isolated from Soncini's protocol. The authors hypothesized that Soncini's protocol isolated cells in an earlier state of stemness (Diaz-Prado et al., 2011). Therefore, it seems that current protocols could potentially fuel simultaneous isolation and cross-contamination and hence are not yet eligible enough to ensure isolation of the desired cells.

### Region of Cell Isolation on Placental Disk

Region at the amniotic membrane from which cells have been isolated may determine the characteristics of the cells (Figure 2). A study demonstrated that cells isolated from placental region had significantly higher mitochondrial activity while significantly fewer reactive oxygen species (ROS) (Banerjee et al., 2015). Centurione et al. isolated cells from four different regions according to their position relative to the umbilical cord. The first area, closer to umbilical cord, was named the central area; the second, in the middle, was considered the intermediate area; the third was named the peripheral area; and the fourth, the reflected area, corresponded to the chorion leave (Centurione et al., 2018). They reported that the peripheral and reflected areas had the highest levels of expression of OCT-4 and SOX-2. On the contrary, the expression of embryonic markers, SSEA-4 and TRA-1-60, was not different among the different areas which indicated homogeneity (Centurione et al., 2018).

### Measuring Methods

Sensitivities of the methods employed to detect markers of hAECs and hAMSCs could also give rise to some discrepancies. A study used and compared immunocytochemistry and flow cytometry for SSEA-4 detection and reported that both hAECs and hAMSCs were positive for SSEA-4 in 100% of tested amnion samples as detected by flowcytometry. However, immunocytochemistry confirmed the expression of SSEA-4 on hAECs as well as hAMSCs only in 40% of samples. The authors hypothesized the higher sensitivity of flowcytometry vs. immunocytochemistry to be a possible explanation for this discrepancy (Bilic et al., 2008). Therefore, sensitivity and specificity of measuring methods should be considered not only upon their application but also while comparing the results of various studies.

The marker expression on gene and protein level should also be considered. A study reported that although Oct-3/4 transcripts were detected hAECs and hAMSCs, its protein was found only in hAECs by immunocytochemistry (Bilic et al., 2008). Therefore, it seems crucial that the level of expression, gene or protein, of a marker need to be considered while reporting comparing the results of studies.

### Gestational Age

Human amniotic membrane is only easily obtainable after childbirth; therefore, there is limited information concerning the phenotypic and functional differences between cells isolated from amniotic membranes preterm and term cesarean sections. Gestational age is thought to have an effect on the expression of pluripotency markers of hAECs including Nanog, SOX2, TRA1-60, and TRA1-81 which have higher expression on hAECs isolated from preterm (17–19 weeks) than term cesarean sections (>37 weeks) (Izumi et al., 2009; Barboni et al., 2014). Although there is limited evidence on the effect of gestational age on the markers expressed on human amniotic cells, there are studies which investigated its effects among zoonotic samples. A study conducted on ovine amniotic epithelial cells reported that cells of amniotic membranes isolated at early stages of pregnancy expressed higher basal and sustained levels of telomerase reverse transcriptase (TERT), SOX2 and Nanog even after *in-vitro* adipogenic differentiation (Barboni et al., 2014). A study reported that there was no expression of TERT mRNA in hAECs isolated form term placenta which could be explained by a progressive switch off during pregnancy (Miki et al., 2005). In addition, telomerase activity in murine amniotic epithelia cells isolated from mid stage amniotic membrane was higher compared to that of the late stages (Nakajima et al., 2001). However, mRNA expression of OCT4 in human was not affected by gestational age (Izumi et al., 2009).

## New Insights and Future Directions

Herein, we reviewed and compared various studies to shed light on the existing discrepancies in characterization of human placenta-derived amniotic epithelial and mesenchymal stromal cells, which could be potentially due to reasons including epithelial to mesenchymal transition, cell heterogeneity, passage number, cross-contamination, region of cell isolation on placental disk, isolation protocols, measuring methods, and gestational age. The potential causes of discrepancies need further consideration prior to the application of these cells in the clinic. As an early step toward overcoming the challenges, some suggestions which could be of potential use in practice are discussed here.

Epithelial to mesenchymal transition could affect the function and marker characterization of the cells both in basic and clinical research. Although some factors involved in EMT have previously been described, including TGF-β (Alcaraz et al., 2013), TNF-α, and matrix metalloproteinases (Janzen et al., 2017), the whole mechanisms of EMT remain unclear to date. EMT needs to be considered for the subcultures aimed to prepare hAECs for research and clinical use. Some studies employed different methods to avoid EMT. A study used xenobiotic-free medium for the culture of hAECs to eliminate the potential effects of growth factors (Pratama et al., 2011). Although the use of EMT-inhibitors could be of value in minimizing the risk of EMT, their potential adverse effects on the cells need to be investigated for safe clinical use. The application of hAECs in their first passage culture for primary cell therapy could keep the occurrence of EMT to a minimum among cells. Nevertheless, the use of the cells in earlier passages, despite being seemingly useful in minimizing EMT, could adversely affect heterogeneity which is another concern involved in the witnessed discrepancies. Heterogeneity decreases during the culture of both hAECs and hAMSCs. In a study, hAECs downregulated dopaminergic markers after seven days of culture, probably through the dedifferentiation process, which resulted in reduced cell heterogeneity (Niknejad et al., 2012). Therefore, defining certain standards of the controlled sub-culture could eliminate heterogeneity as well as EMT.

Simultaneous isolation and cross-contamination of two cell types are among causes of discrepancies which have also been reported in tissues with similar histology to amniotic membrane including cornea with the possibility of simultaneous isolation of endothelial cells with stromal keratocytes, and skin with the possibility of simultaneous isolation of keratinocyte and fibroblasts. Therefore, it is assumed that the methods used to solve the problems in those tissues could be of value in amniotic membrane. A study used antifibroblast magnetic microbeads to deplete the majority of the contaminating corneal fibroblasts (Peh et al., 2012). The skin explant technique, physical agitation with magnetic stirring, density gradient centrifugation, gravity-assisted cell sorting based on a passive filtration of keratinocytes resulted in the propagation of a highly enriched keratinocyte population (Dragunova et al., 2012; Mahabal et al., 2016). It could be suggested that more innovative isolation techniques are required to isolate hAECs form one side of the amniotic membrane and hAMSCs from the other side in a separate manner.

The functional variety of cells isolated from different regions can be considered for further specific clinical use. The cytoplasm of hAECs isolated from the peripheral area contained the highest level of lipid granules. Centurione et al. suggested that this area could be the most capable of immune modulatory effects (Centurione et al., 2018) since the granules have been associated with prostaglandin E secretion by amniotic membrane (Kang et al., 2012; Park et al., 2016). Moreover, hAECs in the central area expressed higher levels of α-fetoprotein compared to other regions. Consequently, an enriched population of cells isolated from this region has the potential to be applied in hepatic differentiation (Centurione et al., 2018). The region of isolation needs to be determined based on the target clinical features of desired cells. Notably, not all regions of the amniotic membrane are suitable for clinical use. The zone of altered morphology, located near the lower uterine pole and cervix, is associated with apoptosis of cells and degradation of basement membrane by matrix metalloproteinases which results in structural weakness and marked disruption of the connective tissue layers and marked reduction of the thickness and cellularity of the amniotic membrane (Peirovi et al., 2012).

Gestational age, another cause for discrepancy, is a clinical term used by obstetricians that is timed from the first day of the last menstrual period in weeks and days. This clinical age differs by approximately 2 weeks from the time of fertilization used by embryologist in basic research. Therefore, the difference in the actual age of the membrane due to various definitions of gestational age could give rise to discrepancy. Therefore, researchers need to make sure that they have the same definition of GA and use it in a united way to avoid discrepancy. It is of importance to notice that preterm amniotic membrane could not be clinically used due to ethical and practical considerations. The amniotic membranes which could be used in the clinic need to be obtained from elective caesarian sections, which are referred to those caesarian sections that are not associated with any medical or surgical indications and have been conducted as per mother's request (Diema Konlan et al., 2019). Normal uncomplicated pregnancies usually reach term and thus preterm placenta of normal pregnancies are not accessible for clinical use. Preterm labors are usually associated with underling diseases or conditions and thus the amniotic membranes obtained from these births are not the clinicians first choice due to possible defects. Noteworthy, epithelial and mesenchymal stromal cells derived from preterm animal placenta is appropriate for mechanistical investigations and research use.

Considering the witnessed discrepancies in the characterization of hAECs and hAMSCs markers and their potential causes along with the promising results of clinical applications of these cells, more research is needed to address the sensitivity and specificity of markers in their characterization as well as determining the most suitable isolation marker(s) for hAMSCs and hAECs characterization. Until optimal approaches for overcoming the potential undesirable effects of above-mentioned causes of discrepancies are achieved, it is suggested that the passage number of cells mentioned in the study, isolation protocols, region of isolation, and gestational age be stated in the articles and any future products with clinical applications.

## Author Contributions

S-HG, MA-K, and HN: conceptualization. SH-G, MA-K, HN, and TT: writing—original draft. HN and SB: writing—review, editing, and supervision. HN: resources.

### Conflict of Interest

The authors declare that the research was conducted in the absence of any commercial or financial relationships that could be construed as a potential conflict of interest.

## References

[B1] Abbasi-Kangevari M. Ghamari S. H. Safaeinejad F. Bahrami S. Niknejad H. (2019). Potential therapeutic features of human amniotic mesenchymal stem cells in multiple sclerosis: immunomodulation, inflammation suppression, angiogenesis promotion, oxidative stress inhibition, neurogenesis induction, MMPs regulation, and remyelination stimulation. Front. Immunol. 10:238. 10.3389/fimmu.2019.00238 30842772 PMC6391358

[B2] Adds P. J. Hunt C. Hartley S. (2001). Bacterial contamination of amniotic membrane. Br. J. Ophthalmol. 85, 228–230. 10.1136/bjo.85.2.228 11159493 PMC1723825

[B3] Ahmed A. S. Sheng M. H. Wasnik S. Baylink D. J. Lau K. W. (2017). Effect of aging on stem cells. World J. Exp. Med. 7, 1–10. 10.5493/wjem.v7.i1.1 28261550 PMC5316899

[B4] Alcaraz A. Mrowiec A. Insausti C. L. Garcia-Vizcaino E. M. Ruiz-Canada C. Lopez-Martinez M. C.. (2013). Autocrine TGF-beta induces epithelial to mesenchymal transition in human amniotic epithelial cells. Cell Transplant. 22, 1351–1367. 10.3727/096368912X657387 23031712

[B5] Alikarami F. Yari F. Amirizadeh N. Nikougoftar M. Jalili M. A. (2015). The immunosuppressive activity of amniotic membrane mesenchymal stem cells on T lymphocytes. Avicenna J. Med. Biotechnol. 7, 90–96. 26306147 PMC4508338

[B6] Alviano F. Fossati V. Marchionni C. Arpinati M. Bonsi L. Franchina M.. (2007). Term amniotic membrane is a high throughput source for multipotent mesenchymal stem cells with the ability to differentiate into endothelial cells *in vitro*. BMC Dev. Biol. 7:11. 10.1186/1471-213X-7-11 17313666 PMC1810523

[B7] Araujo A. B. Furlan J. M. Salton G. D. Schmalfuss T. Rohsig L. M. Silla L. M. R.. (2018). Isolation of human mesenchymal stem cells from amnion, chorion, placental decidua and umbilical cord: comparison of four enzymatic protocols. Biotechnol. Lett. 40, 989–998. 10.1007/s10529-018-2546-z 29619744

[B8] Araujo A. B. Salton G. D. Furlan J. M. Schneider N. Angeli M. H. Laureano A. M.. (2017). Comparison of human mesenchymal stromal cells from four neonatal tissues: amniotic membrane, chorionic membrane, placental decidua and umbilical cord. Cytotherapy 19, 577–585. 10.1016/j.jcyt.2017.03.001 28343898

[B9] Baker E. K. Malhotra A. Lim R. Jacobs S. E. Hooper S. B. Davis P. G.. (2019). Human amnion cells for the prevention of bronchopulmonary dysplasia: a protocol for a phase I dose escalation study. BMJ Open 9:e026265. 10.1136/bmjopen-2018-026265 30826799 PMC6398764

[B10] Banas R. A. Trumpower C. Bentlejewski C. Marshall V. Sing G. Zeevi A. (2008). Immunogenicity and immunomodulatory effects of amnion-derived multipotent progenitor cells. Hum. Immunol. 69, 321–328. 10.1016/j.humimm.2008.04.007 18571002

[B11] Banerjee A. Weidinger A. Hofer M. Steinborn R. Lindenmair A. Hennerbichler-Lugscheider S.. (2015). Different metabolic activity in placental and reflected regions of the human amniotic membrane. Placenta 36, 1329–1332. 10.1016/j.placenta.2015.08.015 26386652

[B12] Barboni B. Russo V. Curini V. Martelli A. Berardinelli P. Mauro A.. (2014). Gestational stage affects amniotic epithelial cells phenotype, methylation status, immunomodulatory and stemness properties. Stem. Cell Rev. 10, 725–741. 10.1007/s12015-014-9519-y 24867872 PMC4167432

[B13] Bilic G. Zeisberger S. M. Mallik A. S. Zimmermann R. Zisch A. H. (2008). Comparative characterization of cultured human term amnion epithelial and mesenchymal stromal cells for application in cell therapy. Cell Transplant. 17, 955–968. 10.3727/096368908786576507 19069637

[B14] Bollini S. Silini A. R. Banerjee A. Wolbank S. Balbi C. Parolini O. (2018). Cardiac restoration stemming from the placenta tree: insights from fetal and perinatal cell biology. Front. Physiol. 9:385. 10.3389/fphys.2018.00385 29695981 PMC5904405

[B15] Brown C. McKee C. Bakshi S. Walker K. Hakman E. Halassy S.. (2019). Mesenchymal stem cells: cell therapy and regeneration potential. J. Tissue Eng. Regen. Med. 13, 1738–1755. 10.1002/term.2914 31216380

[B16] Caruso M. Evangelista M. Parolini O. (2012). Human term placental cells: phenotype, properties and new avenues in regenerative medicine. Int. J. Mol. Cell. Med. 1, 64–74. 24551761 PMC3920494

[B17] Centurione L. Passaretta F. Centurione M. A. Munari S. Vertua E. Silini A.. (2018). Mapping of the human placenta: experimental evidence of amniotic epithelial cell heterogeneity. Cell. Transplant. 27, 12–22. 10.1177/0963689717725078 29562779 PMC6434477

[B18] Chang C. J. Yen M. L. Chen Y. C. Chien C. C. Huang H. I. Bai C. H.. (2006). Placenta-derived multipotent cells exhibit immunosuppressive properties that are enhanced in the presence of interferon-gamma. Stem Cells 24, 2466–2477. 10.1634/stemcells.2006-0071 17071860

[B19] Chen P. M. Lin C. H. Li N. T. Wu Y. M. Lin M. T. Hung S. C.. (2015). c-Maf regulates pluripotency genes, proliferation/self-renewal, and lineage commitment in ROS-mediated senescence of human mesenchymal stem cells. Oncotarget 6, 35404–35418. 10.18632/oncotarget.6178 26496036 PMC4742114

[B20] Covas D. T. Siufi J. L. Silva A. R. Orellana M. D. (2003). Isolation and culture of umbilical vein mesenchymal stem cells. Braz. J. Med. Biol. Res. 36, 1179–1183. 10.1590/S0100-879X2003000900006 12937783

[B21] Diaz-Prado S. Muinos-Lopez E. Hermida-Gomez T. Rendal-Vazquez M. E. Fuentes-Boquete I. de Toro F. J.. (2011). Isolation and characterization of mesenchymal stem cells from human amniotic membrane. Tissue Eng. Part C Methods 17, 49–59. 10.1089/ten.tec.2010.0136 20673138

[B22] Diema Konlan K. Baku E. K. Japiong M. Dodam Konlan K. Amoah R. M. (2019). Reasons for women's choice of elective caesarian section in duayaw nkwanta hospital. J. Pregnancy 2019:2320743. 10.1155/2019/2320743 31360548 PMC6642781

[B23] D'Ippolito G. Diabira S. Howard G. A. Menei P. Roos B. A. Schiller P. C. (2004). Marrow-isolated adult multilineage inducible (MIAMI) cells, a unique population of postnatal young and old human cells with extensive expansion and differentiation potential. J. Cell. Sci. 117, 2971–2981. 10.1242/jcs.01103 15173316

[B24] Dragunova J. Kabat P. Koller J. Jarabinska V. (2012). Experience gained during the long term cultivation of keratinocytes for treatment of burns patients. Cell Tissue Bank. 13, 471–478. 10.1007/s10561-011-9275-z 21847560

[B25] Elwan M. A. Sakuragawa N. (1997). Evidence for synthesis and release of catecholamines by human amniotic epithelial cells. Neuroreport 8, 3435–3438. 10.1097/00001756-199711100-00004 9427302

[B26] Evron A. Goldman S. Shalev E. (2011). Human amniotic epithelial cells cultured in substitute serum medium maintain their stem cell characteristics for up to four passages. Int. J. Stem Cells 4, 123–132. 10.15283/ijsc.2011.4.2.123 24298345 PMC3840962

[B27] Fatimah S. S. Ng S. L. Chua K. H. Hayati A. R. Tan A. E. Tan G. C. (2010). Value of human amniotic epithelial cells in tissue engineering for cornea. Hum. Cell. 23, 141–151. 10.1111/j.1749-0774.2010.00096.x 21166885

[B28] Fatimah S. S. Tan G. C. Chua K. Fariha M. M. Tan A. E. Hayati A. R. (2013). Stemness and angiogenic gene expression changes of serial-passage human amnion mesenchymal cells. Microvasc. Res. 86, 21–29. 10.1016/j.mvr.2012.12.004 23261754

[B29] Garcia-Castro I. L. Garcia-Lopez G. Avila-Gonzalez D. Flores-Herrera H. Molina-Hernandez A. Portillo W.. (2015). Markers of pluripotency in human amniotic epithelial cells and their differentiation to progenitor of cortical neurons. PLoS ONE 10:e0146082. 10.1371/journal.pone.0146082 26720151 PMC4697857

[B30] Garcia-Lopez G. Garcia-Castro I. L. Avila-Gonzalez D. Molina-Hernandez A. Flores-Herrera H. Merchant-Larios H.. (2015). [Human amniotic epithelium (HAE) as a possible source of stem cells (SC)]. Gac. Med. Mex. 151, 66–74. 25739486

[B31] Ge X. Wang I. N. Toma I. Sebastiano V. Liu J. Butte M. J.. (2012). Human amniotic mesenchymal stem cell-derived induced pluripotent stem cells may generate a universal source of cardiac cells. Stem Cells Dev. 21, 2798–2808. 10.1089/scd.2011.0435 22530853 PMC3464077

[B32] Ghasemzadeh M. Hosseini E. Ahmadi M. Kamalizad M. Amirizadeh N. (2018). Comparable osteogenic capacity of mesenchymal stem or stromal cells derived from human amnion membrane and bone marrow. Cytotechnology 70, 729–739. 10.1007/s10616-017-0177-1 29305674 PMC5851966

[B33] Gomez Dominguez R. (2008). Human Amniotic Epithelial Cells: Isolation and Characterisation. Giessen: VVB Laufersweiler Verlag.

[B34] Gotherstrom C. Ringden O. Tammik C. Zetterberg E. Westgren M. Le Blanc K. (2004). Immunologic properties of human fetal mesenchymal stem cells. Am. J. Obstet. Gynecol. 190, 239–245. 10.1016/j.ajog.2003.07.022 14749666

[B35] Gramignoli R. Srinivasan R. C. Kannisto K. Strom S. C. (2016). Isolation of human amnion epithelial cells according to current good manufacturing procedures. Curr. Protoc. Stem Cell Biol. 37, 1e.10.11–11e.10.13. 10.1002/cpsc.2 27171794

[B36] Guarino M. Tosoni A. Nebuloni M. (2009). Direct contribution of epithelium to organ fibrosis: epithelial-mesenchymal transition. Hum. Pathol. 40, 1365–1376. 10.1016/j.humpath.2009.02.020 19695676

[B37] Gupta A. Kedige S. D. Jain K. (2015). Amnion and chorion membranes: potential stem cell reservoir with wide applications in periodontics. Int. J. Biomater. 2015:274082. 10.1155/2015/274082 26770199 PMC4684856

[B38] Hilmy N. Yusof N. Nather A. (eds). (2018). Human Amniotic Membrane: Basic Science and Clinical Application. Singapore: World Scientific.

[B39] Iaffaldano L. Nardelli C. Raia M. Mariotti E. Ferrigno M. Quaglia F.. (2013). High aminopeptidase N/CD13 levels characterize human amniotic mesenchymal stem cells and drive their increased adipogenic potential in obese women. Stem Cells Dev. 22, 2287–2297. 10.1089/scd.2012.0499 23488598 PMC3730375

[B40] Ilancheran S. Michalska A. Peh G. Wallace E. M. Pera M. Manuelpillai U. (2007). Stem cells derived from human fetal membranes display multilineage differentiation potential. Biol. Reprod. 77, 577–588. 10.1095/biolreprod.106.055244 17494917

[B41] In 't Anker P. S. Scherjon S. A. Kleijburg-van der Keur C. Noort W. A. Claas F. H. Willemze R.. (2003). Amniotic fluid as a novel source of mesenchymal stem cells for therapeutic transplantation. Blood 102, 1548–1549. 10.1182/blood-2003-04-1291 12900350

[B42] Insausti C. L. Blanquer M. Garcia-Hernandez A. M. Castellanos G. Moraleda J. M. (2014). Amniotic membrane-derived stem cells: immunomodulatory properties and potential clinical application. Stem Cells Cloning 7, 53–63. 10.2147/SCCAA.S58696 24744610 PMC3969346

[B43] Izumi M. Pazin B. J. Minervini C. F. Gerlach J. Ross M. A. Stolz D. B.. (2009). Quantitative comparison of stem cell marker-positive cells in fetal and term human amnion. J. Reprod. Immunol. 81, 39–43. 10.1016/j.jri.2009.02.007 19501410

[B44] Janzen C. Sen S. Lei M. Y. Gagliardi de Assumpcao M. Challis J. Chaudhuri G. (2017). The role of epithelial to mesenchymal transition in human amniotic membrane rupture. J. Clin. Endocrinol. Metab. 102, 1261–1269. 10.1210/jc.2016-3150 28388726 PMC5460731

[B45] Jin H. Yang Q. Ji F. Zhang Y. J. Zhao Y. Luo M. (2015). Human amniotic epithelial cell transplantation for the repair of injured brachial plexus nerve: evaluation of nerve viscoelastic properties. Neural Regen. Res. 10, 260–265. 10.4103/1673-5374.152380 25883625 PMC4392674

[B46] Kakishita K. Elwan M. A. Nakao N. Itakura T. Sakuragawa N. (2000). Human amniotic epithelial cells produce dopamine and survive after implantation into the striatum of a rat model of Parkinson's disease: a potential source of donor for transplantation therapy. Exp. Neurol 165, 27–34. 10.1006/exnr.2000.7449 10964482

[B47] Kang J. W. Koo H. C. Hwang S. Y. Kang S. K. Ra J. C. Lee M. H.. (2012). Immunomodulatory effects of human amniotic membrane-derived mesenchymal stem cells. J. Vet. Sci. 13, 23–31. 10.4142/jvs.2012.13.1.23 22437532 PMC3317453

[B48] Kim J. Kang H. M. Kim H. Kim M. R. Kwon H. C. Gye M. C.. (2007). *Ex vivo* characteristics of human amniotic membrane-derived stem cells. Cloning Stem Cells 9, 581–594. 10.1089/clo.2007.0027 18154518

[B49] Kitala D. Klama-Baryla A. Kraut M. Labus W. Kawecki M. (2018). Isolation, culturing and preparation for transplantation of amniotic mesenchymal stem cells repetitive and reproducible laboratory, technical protocol. Ann. Biol. Clin. 76, 562–567. 10.1684/abc.2018.1375 30154069

[B50] Koike C. Zhou K. Takeda Y. Fathy M. Okabe M. Yoshida T.. (2014). Characterization of amniotic stem cells. Cell Reprogram 16, 298–305. 10.1089/cell.2013.0090 25068631 PMC4116113

[B51] Kong X. Y. Cai Z. Pan L. Zhang L. Shu J. Dong Y. L.. (2008). Transplantation of human amniotic cells exerts neuroprotection in MPTP-induced Parkinson disease mice. Brain Res. 1205, 108–115. 10.1016/j.brainres.2008.02.040 18353283

[B52] Koyano S. Fukui A. Uchida S. Yamada K. Asashima M. Sakuragawa N. (2002). Synthesis and release of activin and noggin by cultured human amniotic epithelial cells. Dev. Growth Differ. 44, 103–112. 10.1046/j.1440-169x.2002.00626.x 11940097

[B53] Kubo M. Sonoda Y. Muramatsu R. Usui M. (2001). Immunogenicity of human amniotic membrane in experimental xenotransplantation. Invest. Ophthalmol. Vis. Sci. 42, 1539–1546. 11381058

[B54] Lee O. K. Kuo T. K. Chen W. M. Lee K. D. Hsieh S. L. Chen T. H. (2004). Isolation of multipotent mesenchymal stem cells from umbilical cord blood. Blood 103, 1669–1675. 10.1182/blood-2003-05-1670 14576065

[B55] Lefebvre S. Adrian F. Moreau P. Gourand L. Dausset J. Berrih-Aknin S.. (2000). Modulation of HLA-G expression in human thymic and amniotic epithelial cells. Hum. Immunol. 61, 1095–1101. 10.1016/S0198-8859(00)00192-0 11137212

[B56] Lemke A. Castillo-Sanchez J. C. Prodinger F. Ceranic A. Hennerbichler-Lugscheider S. Perez-Gil J.. (2017). Human amniotic membrane as newly identified source of amniotic fluid pulmonary surfactant. Sci. Rep. 7:6406. 10.1038/s41598-017-06402-w 28743969 PMC5527005

[B57] Lim R. Chan S. T. Tan J. L. Mockler J. C. Murphy S. V. Wallace E. M. (2013). Preterm human amnion epithelial cells have limited reparative potential. Placenta 34, 486–492. 10.1016/j.placenta.2013.03.010 23597502

[B58] Lim R. Malhotra A. Tan J. Chan S. T. Lau S. Zhu D.. (2018). First-in-human administration of allogeneic amnion cells in premature infants with bronchopulmonary dysplasia: a safety study. Stem Cells Transl. Med. 7, 628–635. 10.1002/sctm.18-0079 30078207 PMC6127230

[B59] Liu Q. W. Liu Q. Y. Li J. Y. Wei L. Ren K. K. Zhang X. C.. (2018). Therapeutic efficiency of human amniotic epithelial stem cell-derived functional hepatocyte-like cells in mice with acute hepatic failure. Stem Cell Res. Ther. 9:321. 10.1186/s13287-018-1063-2 30463600 PMC6249765

[B60] Lupia M. Angiolini F. Bertalot G. Freddi S. Sachsenmeier K. F. Chisci E.. (2018). CD73 regulates stemness and epithelial-mesenchymal transition in ovarian cancer-initiating cells. Stem Cell Rep. 10, 1412–1425. 10.1016/j.stemcr.2018.02.009 29551673 PMC5998305

[B61] Magatti M. Caruso M. De Munari S. Vertua E. De D. Manuelpillai U.. (2015). Human amniotic membrane-derived mesenchymal and epithelial cells exert different effects on monocyte-derived dendritic cell differentiation and function. Cell Transplant. 24, 1733–1752. 10.3727/096368914X684033 25259480

[B62] Magatti M. De Munari S. Vertua E. Gibelli L. Wengler G. S. Parolini O. (2008). Human amnion mesenchyme harbors cells with allogeneic T-cell suppression and stimulation capabilities. Stem Cells 26, 182–192. 10.1634/stemcells.2007-0491 17901399

[B63] Magatti M. De Munari S. Vertua E. Parolini O. (2012). Amniotic membrane-derived cells inhibit proliferation of cancer cell lines by inducing cell cycle arrest. J. Cell Mol. Med. 16, 2208–2218. 10.1111/j.1582-4934.2012.01531.x 22260183 PMC3822990

[B64] Magatti M. Pianta S. Silini A. Parolini O. (2016). Isolation, culture, and phenotypic characterization of mesenchymal stromal cells from the amniotic membrane of the human term placenta. Methods Mol. Biol. 1416, 233–244. 10.1007/978-1-4939-3584-0_13 27236675

[B65] Magatti M. Vertua E. Cargnoni A. Silini A. Parolini O. (2018). The immunomodulatory properties of amniotic cells: the two sides of the coin. Cell Transplant. 27, 31–44. 10.1177/0963689717742819 29562786 PMC6434482

[B66] Mahabal S. Konala V. B. Mamidi M. K. Kanafi M. M. Mishra S. Shankar K.. (2016). Sequential cultivation of human epidermal keratinocytes and dermal mesenchymal like stromal cells *in vitro*. Cytotechnology 68, 1009–1018. 10.1007/s10616-015-9857-x 25698160 PMC4960150

[B67] Manuelpillai U. Moodley Y. Borlongan C. V. Parolini O. (2011). Amniotic membrane and amniotic cells: potential therapeutic tools to combat tissue inflammation and fibrosis? Placenta 4 (32 Suppl.), S320–325. 10.1016/j.placenta.2011.04.010 21570115

[B68] Mariotti E. Mirabelli P. Abate G. Schiattarella M. Martinelli P. Fortunato G.. (2008). Comparative characteristics of mesenchymal stem cells from human bone marrow and placenta: CD10, CD49d, and CD56 make a difference. Stem Cells Dev. 17, 1039–1041. 10.1089/scd.2008.0212 18713024

[B69] Maymo J. L. Riedel R. Perez-Perez A. Magatti M. Maskin B. Duenas J. L.. (2018). Proliferation and survival of human amniotic epithelial cells during their hepatic differentiation. PLoS ONE 13:e0191489. 10.1371/journal.pone.0191489 29346426 PMC5773201

[B70] Miki T. (2011). Amnion-derived stem cells: in quest of clinical applications. Stem Cell Res. Ther. 2:25. 10.1186/scrt66 21596003 PMC3152995

[B71] Miki T. Lehmann T. Cai H. Stolz D. B. Strom S. C. (2005). Stem cell characteristics of amniotic epithelial cells. Stem Cells 23, 1549–1559. 10.1634/stemcells.2004-0357 16081662

[B72] Miki T. Marongiu F. Dorko K. Ellis E. C. Strom S. C. (2010). Isolation of amniotic epithelial stem cells. Curr. Protoc. Stem Cell Biol. 12, 1E.3.1–1E.3.10. 10.1002/9780470151808.sc01e03s12 20049689

[B73] Miki T. Strom S. C. (2006). Amnion-derived pluripotent/multipotent stem cells. Stem Cell Rev. 2, 133–142. 10.1007/s12015-006-0020-0 17237552

[B74] Moodley Y. Ilancheran S. Samuel C. Vaghjiani V. Atienza D. Williams E. D.. (2010). Human amnion epithelial cell transplantation abrogates lung fibrosis and augments repair. Am. J. Respir. Crit. Care Med. 182, 643–651. 10.1164/rccm.201001-0014OC 20522792

[B75] Motedayyen H. Esmaeil N. Tajik N. Khadem F. Ghotloo S. Khani B.. (2017). Method and key points for isolation of human amniotic epithelial cells with high yield, viability and purity. BMC Res. Notes 10:552. 10.1186/s13104-017-2880-6 29096713 PMC5669002

[B76] Murphy S. Rosli S. Acharya R. Mathias L. Lim R. Wallace E.. (2010). Amnion epithelial cell isolation and characterization for clinical use. Curr. Protoc. Stem Cell Biol. Chapter 1:Unit 1E.6.1–1E.6.25. 10.1002/9780470151808.sc01e06s13 20373516

[B77] Murphy S. V. Kidyoor A. Reid T. Atala A. Wallace E. M. Lim R. (2014). Isolation, cryopreservation and culture of human amnion epithelial cells for clinical applications. J. Vis. Exp. 94:e52085 10.3791/52085 PMC4356357 25548905

[B78] Nakajima T. Enosawa S. Mitani T. Li X. K. Suzuki S. Amemiya H.. (2001). Cytological examination of rat amniotic epithelial cells and cell transplantation to the liver. Cell Transplant. 10, 423–427. 10.3727/000000001783986620 11549066

[B79] Nanbu Y. Fujii S. Konishi I. Nonogaki H. Mori T. (1989). CA 125 in the epithelium closely related to the embryonic ectoderm: the periderm and the amnion. Am. J. Obstet. Gynecol. 161, 462–467. 10.1016/0002-9378(89)90542-5 2669495

[B80] Niknejad H. Deihim T. Ahmadiani A. Jorjani M. Peirovi H. (2012). Permanent expression of midbrain dopaminergic neurons traits in differentiated amniotic epithelial cells. Neurosci. Lett. 506, 22–27. 10.1016/j.neulet.2011.10.038 22037229

[B81] Niknejad H. Yazdanpanah G. Ahmadiani A. (2016). Induction of apoptosis, stimulation of cell-cycle arrest and inhibition of angiogenesis make human amnion-derived cells promising sources for cell therapy of cancer. Cell Tissue Res. 363, 599–608. 10.1007/s00441-016-2364-3 26846225

[B82] Ochsenbein-Kolble N. Bilic G. Hall H. Huch R. Zimmermann R. (2003). Inducing proliferation of human amnion epithelial and mesenchymal cells for prospective engineering of membrane repair. J. Perinat. Med. 31, 287–294. 10.1515/JPM.2003.040 12951883

[B83] Ogawa A. Terada S. Sakuragawa N. Masuda S. Nagao M. Miki M. (2003). Progesterone, but not 17beta-estradiol, up-regulates erythropoietin (EPO) production in human amniotic epithelial cells. J. Biosci. Bioeng. 96, 448–453. 10.1016/S1389-1723(03)70130-3 16233554

[B84] Okazaki T. Honjo T. (2007). PD-1 and PD-1 ligands: from discovery to clinical application. Int. Immunol. 19, 813–824. 10.1093/intimm/dxm057 17606980

[B85] Paracchini V. Carbone A. Colombo F. Castellani S. Mazzucchelli S. Gioia S. D.. (2012). Amniotic mesenchymal stem cells: a new source for hepatocyte-like cells and induction of CFTR expression by coculture with cystic fibrosis airway epithelial cells. J. Biomed. Biotechnol. 2012:575471. 10.1155/2012/575471 22315512 PMC3270433

[B86] Park J. H. Park E. B. Lee J. Y. Min J. Y. (2016). Identification of novel membrane-associated prostaglandin E synthase-1 (mPGES-1) inhibitors with anti-influenza activities *in vitro*. Biochem. Biophys. Res. Commun. 469, 848–855. 10.1016/j.bbrc.2015.11.129 26673392

[B87] Parolini O. Alviano F. Bagnara G. P. Bilic G. Buhring H. J. Evangelista M.. (2008). Concise review: isolation and characterization of cells from human term placenta: outcome of the first international workshop on placenta derived stem cells. Stem Cells 26, 300–311. 10.1634/stemcells.2007-0594 17975221

[B88] Pasquinelli G. Tazzari P. Ricci F. Vaselli C. Buzzi M. Conte R.. (2007). Ultrastructural characteristics of human mesenchymal stromal (stem) cells derived from bone marrow and term placenta. Ultrastruct. Pathol. 31, 23–31. 10.1080/01913120601169477 17455095

[B89] Peh G. S. Lee M. X. Wu F. Y. Toh K. P. Balehosur D. Mehta J. S. (2012). Optimization of human corneal endothelial cells for culture: the removal of corneal stromal fibroblast contamination using magnetic cell separation. Int. J. Biomater. 2012:601302. 10.1155/2012/601302 22287967 PMC3263628

[B90] Peirovi H. Rezvani N. Hajinasrollah M. Mohammadi S. S. Niknejad H. (2012). Implantation of amniotic membrane as a vascular substitute in the external jugular vein of juvenile sheep. J. Vasc. Surg. 56, 1098–1104. 10.1016/j.jvs.2012.02.036 22560305

[B91] Peric Z. Skegro I. Durakovic N. Desnica L. Pulanic D. Serventi-Seiwerth R.. (2018). Amniotic membrane transplantation-a new approach to crossing the HLA barriers in the treatment of refractory ocular graft-versus-host disease. Bone Marrow Transplant. 53, 1466–1469. 10.1038/s41409-018-0140-6 30089899

[B92] Phermthai T. Thongbopit S. Pokathikorn P. Wichitwiengrat S. Julavijitphong S. Tirawanchai N. (2017). Carcinogenicity, efficiency and biosafety analysis in xeno-free human amniotic stem cells for regenerative medical therapies. Cytotherapy 19, 990–1001. 10.1016/j.jcyt.2017.04.004 28566211

[B93] Pittenger M. F. Mackay A. M. Beck S. C. Jaiswal R. K. Douglas R. Mosca J. D.. (1999). Multilineage potential of adult human mesenchymal stem cells. Science 284, 143–147. 10.1126/science.284.5411.143 10102814

[B94] Pogozhykh O. Pogozhykh D. Neehus A. L. Hoffmann A. Blasczyk R. Muller T. (2015). Molecular and cellular characteristics of human and non-human primate multipotent stromal cells from the amnion and bone marrow during long term culture. Stem Cell Res. Ther. 6:150. 10.1186/s13287-015-0146-6 26297012 PMC4546288

[B95] Portmann-Lanz C. B. Schoeberlein A. Huber A. Sager R. Malek A. Holzgreve W.. (2006). Placental mesenchymal stem cells as potential autologous graft for pre- and perinatal neuroregeneration. Am. J. Obstet. Gynecol. 194, 664–673. 10.1016/j.ajog.2006.01.101 16522395

[B96] Pozzobon M. Piccoli M. De Coppi P. (2014). Stem cells from fetal membranes and amniotic fluid: markers for cell isolation and therapy. Cell Tissue Bank. 15, 199–211. 10.1007/s10561-014-9428-y 24554400

[B97] Prakoeswa C. R. S. Natallya F. R. Harnindya D. Thohiroh A. Oktaviyanti R. N. Pratiwi K. D.. (2018). The efficacy of topical human amniotic membrane-mesenchymal stem cell-conditioned medium (hAMMSC-CM) and a mixture of topical hAMMSC-CM + vitamin C and hAMMSC-CM + vitamin E on chronic plantar ulcers in leprosy:a randomized control trial. J. Dermatol. Treat 29, 835–840. 10.1080/09546634.2018.1467541 29671368

[B98] Pratama G. Vaghjiani V. Tee J. Y. Liu Y. H. Chan J. Tan C.. (2011). Changes in culture expanded human amniotic epithelial cells: implications for potential therapeutic applications. PLoS ONE 6:e26136. 10.1371/journal.pone.0026136 22073147 PMC3206797

[B99] PUPS and Shanghai iCELL Biotechnology Co., Ltd, Shanghai, China (2018). hAECs Are Preliminarily Applied in Allogeneic Hematopoietic Stem Cell Transplantation. Available online at: https://ClinicalTrials.gov/show/NCT03759899

[B100] Rennie K. Gruslin A. Hengstschlager M. Pei D. Cai J. Nikaido T.. (2012). Applications of amniotic membrane and fluid in stem cell biology and regenerative medicine. Stem Cells Int. 2012:721538. 10.1155/2012/721538 23093978 PMC3474290

[B101] Roubelakis M. G. Trohatou O. Anagnou N. P. (2012). Amniotic fluid and amniotic membrane stem cells: marker discovery. Stem Cells Int. 2012:107836. 10.1155/2012/107836 22701492 PMC3372280

[B102] Sakuragawa N. Enosawa S. Ishii T. Thangavel R. Tashiro T. Okuyama T.. (2000). Human amniotic epithelial cells are promising transgene carriers for allogeneic cell transplantation into liver. J. Hum. Genet. 45, 171–176. 10.1007/s100380050205 10807543

[B103] Sakuragawa N. Kakinuma K. Kikuchi A. Okano H. Uchida S. Kamo I.. (2004). Human amnion mesenchyme cells express phenotypes of neuroglial progenitor cells. J. Neurosci. Res. 78, 208–214. 10.1002/jnr.20257 15378611

[B104] Sakuragawa N. Misawa H. Ohsugi K. Kakishita K. Ishii T. Thangavel R.. (1997). Evidence for active acetylcholine metabolism in human amniotic epithelial cells: applicable to intracerebral allografting for neurologic disease. Neurosci. Lett. 232, 53–56. 10.1016/s0304-3940(97)00570-3 9292890

[B105] Sakuragawa N. Thangavel R. Mizuguchi M. Hirasawa M. Kamo I. (1996). Expression of markers for both neuronal and glial cells in human amniotic epithelial cells. Neurosci. Lett. 209, 9–12. 10.1016/0304-3940(96)12599-4 8734897

[B106] Samsonraj R. M. Raghunath M. Nurcombe V. Hui J. H. van Wijnen A. J. Cool S. M. (2017). Concise review: multifaceted characterization of human mesenchymal stem cells for use in regenerative medicine. Stem Cells Transl. Med. 6, 2173–2185. 10.1002/sctm.17-0129 29076267 PMC5702523

[B107] Sarvandi S. S. Joghataei M. T. Parivar K. Khosravi M. Sarveazad A. Sanadgol N. (2015). *In vitro* differentiation of rat mesenchymal stem cells to hepatocyte lineage. Iran. J. Basic Med. Sci. 18, 89–97. 25810881 PMC4366749

[B108] Schmelzer E. McKeel D. T. Gerlach J. C. (2019). Characterization of human mesenchymal stem cells from different tissues and their membrane encasement for prospective transplantation therapies. Biomed. Res. Int. 2019:6376271. 10.1155/2019/6376271 30941369 PMC6421008

[B109] Shu J. Guo L. L. Zhang K. H. Cai Z. Cheng L. M. Li R. Q.. (2011). [Experimental study on the induced differentiation of human amnion mesenchymal cells into osteoblasts]. Zhonghua Zheng Xing Wai Ke Za Zhi 27, 362–367. 22259988

[B110] Si J. Dai J. Zhang J. Liu S. Gu J. Shi J.. (2015). Comparative investigation of human amniotic epithelial cells and mesenchymal stem cells for application in bone tissue engineering. Stem Cells Int. 2015:565732. 10.1155/2015/565732 25834575 PMC4365333

[B111] Sivasubramaniyan K. Harichandan A. Schumann S. Sobiesiak M. Lengerke C. Maurer A.. (2013). Prospective isolation of mesenchymal stem cells from human bone marrow using novel antibodies directed against Sushi domain containing 2. Stem Cells Dev. 22, 1944–1954. 10.1089/scd.2012.0584 23406305

[B112] Soncini M. Vertua E. Gibelli L. Zorzi F. Denegri M. Albertini A.. (2007). Isolation and characterization of mesenchymal cells from human fetal membranes. J. Tissue Eng. Regen. Med. 1, 296–305. 10.1002/term.40 18038420

[B113] Spitzhorn L. S. Rahman M. S. Schwindt L. Ho H. T. Wruck W. Bohndorf M.. (2017). Isolation and molecular characterization of amniotic fluid-derived mesenchymal stem cells obtained from caesarean sections. Stem Cells Int. 2017:5932706. 10.1155/2017/5932706 29225627 PMC5684599

[B114] Tabatabaei M. Mosaffa N. Nikoo S. Bozorgmehr M. Ghods R. Kazemnejad S.. (2014). Isolation and partial characterization of human amniotic epithelial cells: the effect of trypsin. Avicenna J. Med. Biotechnol. 6, 10–20. 24523953 PMC3895574

[B115] Takahashi N. Enosawa S. Mitani T. Lu H. Suzuki S. Amemiya H.. (2002). Transplantation of amniotic epithelial cells into fetal rat liver by *in utero* manipulation. Cell Transplant. 11, 443–449. 10.3727/000000002783985602 12382671

[B116] Takahashi S. Ohsugi K. Yamamoto T. Shiomi M. Sakuragawa N. (2001). A novel approach to *ex vivo* gene therapy for familial hypercholesterolemia using human amniotic epithelial cells as a transgene carrier. Tohoku. J. Exp. Med. 193, 279–292. 10.1620/tjem.193.279 11453536

[B117] Tamagawa T. Ishiwata I. Ishikawa H. Nakamura Y. (2008). Induced *in-vitro* differentiation of neural-like cells from human amnion-derived fibroblast-like cells. Hum. Cell 21, 38–45. 10.1111/j.1749-0774.2008.00049.x 18397473

[B118] The Second Affiliated Hospital of Chongqing Medical University Shanghai iCELL Biotechnology Co., Ltd, Shanghai, China (2017). Human Amniotic Epithelial Cells for Asherman's Syndrome. Available online at: https://ClinicalTrials.gov/show/NCT03223454

[B119] Topoluk N. Hawkins R. Tokish J. Mercuri J. (2017). Amniotic mesenchymal stromal cells exhibit preferential osteogenic and chondrogenic differentiation and enhanced matrix production compared with adipose mesenchymal stromal cells. Am. J. Sports Med. 45, 2637–2646. 10.1177/0363546517706138 28541092 PMC5832055

[B120] Tsuji T. Ibaragi S. Hu G. F. (2009). Epithelial-mesenchymal transition and cell cooperativity in metastasis. Cancer Res. 69, 7135–7139. 10.1158/0008-5472.CAN-09-1618 19738043 PMC2760965

[B121] Uchida S. Inanaga Y. Kobayashi M. Hurukawa S. Araie M. Sakuragawa N. (2000). Neurotrophic function of conditioned medium from human amniotic epithelial cells. J. Neurosci. Res. 62, 585–590. 10.1002/1097-4547(20001115)62:4<585::AID-JNR13>3.0.CO;2-U 11070502

[B122] Wei J. P. Zhang T. S. Kawa S. Aizawa T. Ota M. Akaike T.. (2003). Human amnion-isolated cells normalize blood glucose in streptozotocin-induced diabetic mice. Cell Transplant. 12, 545–552. 10.3727/000000003108747000 12953929

[B123] Wolbank S. Peterbauer A. Fahrner M. Hennerbichler S. van Griensven M. Stadler G.. (2007). Dose-dependent immunomodulatory effect of human stem cells from amniotic membrane: a comparison with human mesenchymal stem cells from adipose tissue. Tissue Eng. 13, 1173–1183. 10.1089/ten.2006.0313 17518752

[B124] Wu W. Lan Q. Lu H. Xu J. Zhu A. Fang W.. (2014). Human amnion mesenchymal cells negative co-stimulatory molecules PD-L1 expression and its capacity of modulating microglial activation of CNS. Cell Biochem. Biophys. 69, 35–45. 10.1007/s12013-013-9763-9 24096708

[B125] Yamahara K. Hamada A. Soma T. Okamoto R. Okada M. Yoshihara S.. (2019). Safety and efficacy of amnion-derived mesenchymal stem cells (AM01) in patients with steroid-refractory acute graft-versus-host disease after allogeneic haematopoietic stem cell transplantation: a study protocol for a phase I/II Japanese trial. BMJ Open 9:e026403. 10.1136/bmjopen-2018-026403 31289066 PMC6615811

[B126] Yazdanpanah G. Paeini-Vayghan G. Asadi S. Niknejad H. (2015). The effects of cryopreservation on angiogenesis modulation activity of human amniotic membrane. Cryobiology 71, 413–418. 10.1016/j.cryobiol.2015.09.008 26432457

[B127] Zhou K. Koike C. Yoshida T. Okabe M. Fathy M. Kyo S.. (2013). Establishment and characterization of immortalized human amniotic epithelial cells. Cell Reprogram 15, 55–67. 10.1089/cell.2012.0021 23298399 PMC3567704

